# Correlation Between Screen Time and Autistic Symptoms as Well as Development Quotients in Children With Autism Spectrum Disorder

**DOI:** 10.3389/fpsyt.2021.619994

**Published:** 2021-02-16

**Authors:** Han-Yu Dong, Bing Wang, Hong-Hua Li, Xiao-Jing Yue, Fei-Yong Jia

**Affiliations:** Department of Developmental and Behavioral Pediatrics, The First Hospital of Jilin University, Changchun, China

**Keywords:** screen time, autism spectrum disorder, ABC, CARS, development quotient, Gesell

## Abstract

**Background:** Electronic screen media play an increasingly vital role in children's entertainment; however, excessive screen time may negatively influence child development. The purpose of this study was to investigate the relationship between the screen time of children with autism spectrum disorder (ASD) and their autistic symptoms and development quotients (DQs).

**Methods:** We compared the screen time of 101 children with ASD and 57 typically developing (TD) children. Then, we performed a correlation analysis to determine the correlations between the screen time and the ASD-related scale scores and developmental quotients of the Gesell Developmental Schedules (GDS) of ASD children. We further divided the ASD group into subgroups according to the screen time and age and then separately conducted the above correlation analyses by subgroup.

**Result:** The results showed that the screen time of the children with ASD was longer than that of the TD children (3.34 ± 2.64 h vs. 0.91 ± 0.93 h). The screen time of the children with ASD was positively correlated with the Childhood Autism Rating Scale (CARS) score (*r* = 0.242, *P* = 0.021) and “taste, smell and touch” item of CARS(*r* = 0.304, *P* = 0.005), and negatively correlated with the language DQ of the GDS (*r* = −0.236, *P* = 0.047). The subgroup analysis showed that in the longer screen time subgroup of ASD children, the screen time was positively correlated with the CARS score (*r* = 0.355, *P* = 0.026) and negatively correlated with the DQs of all domains of the GDS (*P* < 0.05). In addition, in the younger age group of ASD children, the screen time was positively correlated with the CARS score (*r* = 0.314, *P* = 0.021) and negatively correlated with the DQs of all domains of the GDS, except for the personal-social behavior domain (*P* < 0.05).

**Conclusion:** Compared with TD children, children with ASD have a longer screen time. The screen time is related to autism-like symptoms and the DQs of children with ASD. The longer the screen time, the more severe the symptoms of ASD (especially sensory symptoms), and the more obvious the developmental delay, especially in ASD children with a longer screen time and younger age, particularly in the language domain.

## Introduction

Autism spectrum disorder (ASD) is a type of neurodevelopmental disorder characterized by persistent deficits in social communication and interaction and stereotyped or repetitive patterns of behavior, interests or activities. The prevalence of autism is increasing yearly, and the latest report indicates that the prevalence of autism is 1/54 individuals (1). Autism is a serious disease that affects children's social adaptability.

The level of early childhood development is related to many factors, including prenatal and postnatal factors. The postnatal factors include biological factors and social-economic factors. In recent years, the widespread use of electronic screens seems to have played a significant role in early childhood development and, may be considered a social-economic factor. Electronic screen media play an increasingly vital role in the entertainment of not only typically developing (TD) children but also children with neurodevelopmental disabilities. So-called screen time mainly consists of time spent watching television or using a computer, tablet computer, or smart mobile phone (2). Children usually spend their screen time watching cartoons and movies, playing video games, using social media and, occasionally, learning (3).

The current situation of children's screen time from the toddler years to adolescence is extremely severe. Studies have shown that a large proportion of very young children living in Australia were exposed to screens for >2 h per day and that 40% of infants aged under 18 months used screens for longer than the time specified in the current recommended guidelines (4). The average daily screen time of preschool children in the United States was found to be as high as 4.1 h, and more than half of children had a daily video time >2 h (5). The Active Healthy Kids Global Alliance surveyed children and youth in 42 countries across 6 continents (representing 60% of the world's population) (6). These findings of children and youth revealed that 62% of 13-year-olds and 63% of 15-year-olds watched over 2 h of television per day on weekdays and weekends (6). The US and European countries as well as Asian countries face this problem. A study in Japan reported that 29.4% of children aged under 18 months and 24.5% of children aged under 30 months watched TV for more than 4 h a day (7). In China, a study involving 27,200 children aged 3–6 years showed that the average daily screen time of preschool children was 1.81 h and that the percentage of children who had >1 h/d of screen time was 62.4% (8). A survey of 20,324 kindergarten children aged 3–4 years in 2018 showed that the average screen time of preschoolers in Shanghai was 170.2 min/day and that 78.6% of children had an average screen time of more than 1 h per day (9). The latest study in Shandong Province in China in 2019 found that the average daily screen time of 2,131 children aged 4–6 years was over 1.5 h. Research has also explored the age of children's first exposure to electronic devices. The average age is 2.25 years, and the youngest age at first exposure to electronic devices is 3 months (0.25 years). Studies have also analyzed the screen time of children in different age groups. The average daily electronic screen time of children aged 4–5 years is 1.52 ± 1.24 h. The two results above both exceed the American Academy of Pediatrics (AAP) recommendations (10).

Excessive screen time has several negative effects in the early development of children. Extended screen time does much more harm than good with negative physiological, cognitive, social, emotional, and legal consequences (11, 12). A long screen time may be linked to negative outcomes in child development, such as poor academic performance (13, 14), obesity (16), and sleep problems (17, 18). Studies have reported that excessive screen time may also lead to social behavior deficits (7) or attention problems (19, 20), especially during critical periods of development. Based on the above reasons, the AAP also recommends that parents avoid exposing children younger than 2 years to digital devices and limit the screen time of children aged 2–5 years to 1 h per day (10). In addition, a longer screen time is associated with poorer performance on developmental tests; a 2019 study of 2,441 children in Canada showed that higher levels of screen time at 24 and 36 months were significantly associated with poorer performance on developmental screening tests at 36 and 60 months. The study provided strong evidence of the correlation between screen time and child development (21). The problem of screen exposure plays an important role in child development.

Studies investigating screen time in children with ASD have also been reported in the literature. Healy et al. found that children with ASD had a longer screen time and less physical activity than TD children (22). In addition, because of their lower activity rate and longer screen time, children with ASD were more commonly overweight or obese than TD children (23). Must et al. reported that children with ASD spent more time in sedentary pursuits than TD children and that the major component driving sedentary behavior was screen time (2). Nevertheless, not all studies reached the same conclusions. Montes conducted a large sample study involving 1,393 children with ASD and 64,163 TD children and concluded that half of US children aged 6–17 years exceeded the AAP-recommended screen time and that children with ASD had amounts of total screen time similar to those of children without ASD. However, children with ASD constituted a higher proportion of children who were high screen users (>2 h/day) (24).

Wu X demonstrated that screen time might be correlated with some autistic-like symptoms (25), but few studies focused on the correlation between screen time and the developmental levels of children with ASD. Therefore, our study compares the screen time of TD children with that of children with ASD to determine whether children with autism have a longer screen time. Then, we explored the correlation between the screen time and the autistic symptoms and development quotients (DQs) of children with ASD.

## Methods

### Participants

In total, 101 children with ASD who were diagnosed for the first time in the Department of Developmental and Behavioral Pediatrics of the First Hospital of Jilin University between November 2019 and August 2020 were selected as the ASD group. The age of the ASD children was 39.33 ± 17.4 months. During the same period, 57 neurotypical children who underwent regular physical examinations in our outpatient department were recruited as the TD group. The age of the TD children was 40.64 ± 20.0 months. The inclusion criterion for the children with ASD was that the ADOS-2 was used to diagnose the children for the first time without systemic intervention. The inclusion criteria for the children with typical development were as follows: children undergoing routine physical examinations in outpatient clinics; children with height and weight development within the normal range; children with a DQ above 85 in all domains of the Gesell Development Schedule (GDS); and children whose outpatient doctors' mental examination clearly indicated that they do not meet the ASD or other neurodevelopmental disorders' diagnostic criteria in the DSM-5. Children with severe physical disability, cardiopulmonary disease or uncontrolled epilepsy were excluded. The study protocol was approved by the ethics committee of our hospital, and informed consent was provided by the parents or caregivers of the children who participated.

### Procedure

We collected information regarding the average daily screen time and general information, including sex, age, height, and weight, from the two groups of children. The children in the ASD group were assessed using the Autism Behavior Checklist (ABC), Childhood Autism Rating Scale (CARS), and GDS. The children with typical development were assessed using only the GDS. We compared the general information and screen time of the ASD group with those of the TD group. Then, we analyzed the correlation between the screen time and the ASD-related scale scores and DQs of the GDS in the ASD group. We further divided the ASD group into subgroups according to the screen time and age. Then, we separately analyzed the correlations between the screen time and the ASD-related scale scores and DQs of the GDS by subgroup.

All scale assessments, background information investigations, and developmental tests were performed by systematically trained assessors with good consistency. All clinical data are uniformly managed by a physician in our research group.

### Screen Time Survey

After the children completed the Gesell Developmental Schedule, the evaluator investigated the children's screen time. In our study, screen time measures the total amount of time that the children were exposed to various electronic screens, such as watching TV, using computers, playing games, and using mobile phones, during the week before the survey. We investigated the screen time on weekdays (Monday to Friday) and weekends (Saturday and Sunday). The evaluator calculated and recorded the average daily screen time of the children using the following formula: average daily screen time (h) = [screen time per day on weekdays (h) × 5 + screen time per day on weekends (h) × 2]/7.

The GDS is currently a very widely used neurodevelopmental scale with good reliability and validity (26, 27). The GDS divides children's neurodevelopment into five domains, namely, adaptability, gross motor, fine motor, language and personal-social. The developmental quotient of the five domains is used to evaluate the level of neurodevelopment. The developmental age (DA) was derived from the tests, and the chronological age (CA) was calculated as the date of assessment minus the date of birth as follows: DQs = DA × 100/CA (28). The higher the DQ, the better the neurodevelopment.

The symptom evaluation scales included the ABC and the CARS. The ABC is a 57-item screening checklist for autism containing 5 subscales (i.e., body behavior, sensory, self-care, language and social interaction). The ABC is designed for parent interviews. The CARS was developed by Schopler and Reichler et al. and used as a diagnostic scale. The CARS consists of 15 scales, and each scale is scored on a continuum from normal to severely abnormal. The CARS requires observation of the performance of ASD children in a consulting room. The reliability and validity of the ABC and CARS are sufficiently good in China (29), reflecting the scales' usefulness for clinical diagnosis and evaluation of ASD symptoms.

### Statistical Methods

SPSS 23.0 statistical software was used for the statistical analysis. The means ± standard deviations of the two groups' basic data were normally distributed. Two independent-sample *t*-tests were used for the comparisons between the groups. The enumeration data are presented as *N* (%), and the differences between the two groups were measured by chi-square tests. A Pearson linear correlation analysis was used to analyze the two normally distributed variables, and a Spearman rank correlation analysis was used to analyze the two nonnormally distributed variables.

## Results

### Demographic Data and Screen Time in the ASD Group and TD Group

There were no differences in sex, age or body mass index (BMI) between the ASD group and the TD group (*P* = 0.617, 0.669, and 0.113, respectively). The screen time in the ASD group was significantly longer than that in the TD group (3.34 ± 2.64 h/day vs. 0.91 ± 0.93 h/day, *P* = 0.001). The details are shown in Table 1.

**Table 1:** Comparison of sex, age, BMI, and screen time between the two groups.

**Group**	**ASD group**	**TD group**	***t*/χ2**	***P***
	***N* = 101**	***N* = 57**		
**Sex**				
Male	78 (77.2%)	42 (73.7%)	0.250	0.617
Female	23 (22.8%)	15 (26.3%)		
Age (m)	39.33 ± 17.4	40.64 ± 20.0	−0.428	0.669
BMI	16.70 ± 2.25	16.14 ± 1.97	1.597	0.113
Screen time (h/day)	3.34 ± 2.64	0.91 ± 0.93	6.646	0.001^*^

* *P < 0.05*.

### Correlations Between Screen Time and ASD Symptoms and DQs in Children With ASD

The results of the Pearson rank correlation test showed that the screen time of the children with ASD was positively correlated with the total score of CARS (*r* = 0.242, *P* = 0.021) and “taste, smell and touch” item of CARS(*r* = 0.304, *P* = 0.005), and negatively correlated with the language DQ (*r* = −0.236, *P* = 0.047). The screen time of the children with ASD was not correlated with the ABC score (*r* = 0.107, *P* = 0.316) and other domains (adaptive behavior, gross motor, fine motor and personal-social behavior) (*r* = −0.051, 0.022, −0.190, and −0.057, respectively, *P* = 0.672, 0.856, 0.113, and 0.642, respectively) of GDS. The details are shown in Table 2.

Table 2Correlations between screen time and ASD symptoms and development quotients.

**Table:** 

	**ABC**	**CARS**	**Adaptive behavior**	**Gross motor**	**Fine motor**	**Language**	**Personal-social behavior**
**A: Correlations between screen time and ABC, CARS score as well as DQs of GDS**							
*r*	0.107	0.242	−0.051	0.022	−0.190	−0.236	−0.057
*P*	0.316	0.021^*^	0.672	0.856	0.113	0.047^*^	0.642

**Table:** 

	***r***	***P***		***r***	***P***
**B: Correlations between screen time and 15 items of CARS scale**					
Relating to people	−0.019	0.862	Taste, smell, touch	0.304	0.005^*^
Imitation	0.002	0.987	Fear or nervous	−0.103	0.357
Emotional response	0.088	0.430	Verbal communication	0.116	0.301
Body use	0.099	0.375	Nonverbal communication	0.176	0.114
Object use	0.050	0.656	Activity level	−0.005	0.962
Adaptation To change	0.092	0.413	Consistency of intellectual response	0.018	0.871
Visual response	0.113	0.314	General impression	0.142	0.204
Listening response	−0.020	0.857			

* *P < 0.05*.

We divided the ASD group into a longer screen time subgroup and a shorter screen time subgroup based on the mean screen time (3.34 h/d) of the children with ASD. The longer screen time subgroup was defined as ST ≥ 3.34 h/day, and the shorter screen time subgroup was defined as ST < 3.34 h/day. The longer screen time subgroup included 44 ASD children, and the average screen time was 5.81 ± 1.93 h/d. The shorter screen time subgroup included 57 ASD children, and the average screen time was 1.43 ± 1.02 h/d. We calculated the correlations between the screen time and the ASD-related scale scores and DQs in the two subgroups of children with ASD. The results showed that among the children with ASD with a screen time longer than 3.34 h per day, the screen time was positively correlated with the CARS score (*r* = 0.355, *P* = 0.026) and negatively correlated with the DQs of all domains of the GDS (see details in Table 3). However, we did not find any correlation between the screen time and the ASD-related scale scores or the DQs in the shorter screen time subgroup.

**Table 3:** Correlations between screen time and ASD-related scale scores and development quotients in the longer and shorter screen time subgroups.

	**Longer screen time subgroup (≥3.34 h/day)**		**Shorter screen time subgroup (<3.34 h/day)**	
	***r***	***P***	***r***	***P***
ABC score	−0.022	0.897	0.168	0.228
CARS score	0.355	0.026^*^	0.182	0.197
Adaptive behavior development quotient	−0.349	0.047^*^	0.192	0.247
Gross motor development quotient	−0.460	0.007^*^	0.297	0.070
Fine motor development quotient	−0.705	0.000^*^	0.306	0.062
Language development quotient	−0.375	0.031^*^	0.010	0.951
Personal-social behavior development quotient	−0.361	0.039^*^	0.195	0.247

* *P < 0.05*.

In addition, we divided the ASD group into an older group and a younger group according to the average age (39 months) to determine whether the screen time was correlated with the ASD symptoms and DQs in these two subgroups. The older subgroup was defined as an age ≥ 39 m, and the younger subgroup was defined as an age <39 m. The older subgroup included 48 ASD children, and the average age was 56.46 ± 16.45 m. The younger subgroup included 53 ASD children, and the average age was 29.00 ± 6.07 m. The results showed that in the younger subgroup, screen time was positively correlated with the CARS score (*r* = 0.314, *P* = 0.021) and negatively correlated with the adaptive behavior (*r* = −0.301, *P* = 0.040), gross motor (*r* = −0.307, *P* = 0.036), fine motor (*r* = −0.335, *P* = 0.021) and language (*r* = −0.386, *P* = 0.007) DQs of the GDS (see details in Table 4). However, we did not find any correlation between the screen time and the ASD-related scale scores or DQs in the older subgroup.

**Table 4:** Correlations between screen time and ASD-related scale scores and development quotients in the older and younger subgroups.

	**Older subgroup (≥39 m)**		**Younger subgroup (<39 m)**	
	***r***	***P***	***r***	***P***
ABC score	−0.081	0.632	0.131	0.351
CARS score	0.019	0.911	0.314	0.021^*^
Adaptive behavior development quotient	0.232	0.276	−0.301	0.040^*^
Gross motor development quotient	0.381	0.066	−0.307	0.036^*^
Fine motor development quotient	−0.101	0.640	−0.335	0.021^*^
Language development quotient	0.117	0.585	−0.386	0.007^*^
Personal-social behavior development quotient	0.323	0.123	−0.274	0.065

* *P < 0.05*.

## Discussion

The main findings of this study are as follows: 1. the screen time of children with ASD was longer than that of TD children, and 2. The screen time was related to the autistic symptoms and DQs of the children with ASD.

### The Screen Time of the Children With ASD Was Longer Than That of the TD Children

A study conducted by Chonchaiya et al. in Thailand in 2011 showed that children with autism watched TV earlier and more frequently than TD children (30). The average age when children start watching TV was 6.44 months, and the average screen time was 4.60 h/d. Mazurek et al. studied the screen time of children with ASD aged 8–18 years and compared with that of their TD siblings and found that the average screen time of the ASD children was 4.5 h/d, which was significantly longer than that of their siblings (15). Other studies have reached similar conclusions. As previously mentioned, the problem of excessive screen time among ASD children is very serious.

In our study, we found that the screen time in the ASD group was significantly longer than that in the TD group. The mean screen time of the children with ASD was approximately 3.34 ± 2.64 h/day; nevertheless, the screen time of the TD children was only 0.91 ± 0.93 h/day, which is consistent with the results of most previous studies. Although many studies have reported that a longer screen time is associated with children with ASD being overweight compared to TD children (31), we did not observe that the children with ASD had a greater BMI than the children with TD. The screen time of ASD children is excessive, causing them to spend less time engaging in outdoor activities than normal children, which may be a reason for the high BMI of ASD children. However, our study did not observe differences in BMI between the children with TD and children with ASD. The reasons may be as follows. First, we did not investigate the children's outdoor activities. Second, all children in this region have limited access to outdoor activities, which may lead to greater homogeneity in BMI. The study province is located in northeastern China, which has high latitudes, short winter sunshine hours, cold weather, and low temperatures and is not suitable for children's outdoor activities. Thus, the lack of a difference in outdoor activity time may explain the lack of a significant difference in BMI between the children with ASD and the TD children. Therefore, such investigations should be carried out in further studies with expanded sample sizes.

### Screen Time Was Related to Autistic Symptoms

Adverse environmental factors constitute an important factor affecting the clinical symptoms of children with ASD. Excessive screen time has adverse effects on children's social participation and behavior regulation (32, 33). Yousef's study found that a screen time > 2 h/d among school-age children was positively correlated with children's autism-like symptoms (34). Our results showed that the screen time was positively correlated with the CARS score. The CARS is a standardized diagnostic scale that, to some extent, reflects the severity of autism-like symptoms. Our results demonstrate that the longer the screen time, the more obvious the autistic symptoms. We did not observe a correlation between the screen time and the ABC scale score, which may be due to the bias of the ABC as a parent-reported scale. All patients included in our study were newly diagnosed. Parents usually visit a doctor due to their child's language impairment and are often unwilling to admit social and autism-related issues. In addition, many families have elderly family members and nannies as the main caregivers of children. Sometimes, parents' knowledge of their children's performance is limited, resulting in bias in the ABC scale. As a result, the ABC scale score might be low and not completely and accurately reflect autism symptoms. The CARS scale is completed by professionals based on observations and can avoid this problem.

In addition, we conducted correlation study on the screen time of children with ASD and the 15 items of the CARS scale, in order to further observe which aspect of the symptoms of children with ASD is affected by the screen exposure. The results showed that there is a positive correlation between the screen time of children with ASD and the score of “taste, smell and touch” item. Till now, no research has been done on this subject. It is speculated that the reasons may be as follows: First, this phenomenon is related to abnormal sensory development in children with autism, and children with obvious sensory abnormalities are more likely to be fond of the visual stimulation brought by screen exposure. Second, it may be due to the long exposure time of the screen, which caused the abnormal development of its sensory pathways and neurodevelopment. However, this is a cross-sectional study, and causality cannot be inferred. In the future, we look forward to a related cohort study to clarify it.

The subgroup analysis showed that the correlation between the screen time and the CARS score was statistically significant in the subgroups with a longer screen time and subgroups with a younger age, while no statistical correlation was observed in the subgroups with a shorter screen time and an older age. This result is consistent with our expectations. ASD is closely related to brain development. Studies have shown that during the early postnatal period or even before birth, the brain of ASD children has multiple neurological defects. The brain undergoes rapid development early after birth and is influenced by the developmental environment (35–37). Therefore, we speculate that the longer children with autism are exposed to unfavorable environmental factors, the lower their brain maturity, and the more obvious their developmental delay and autistic symptoms. Obviously, a longer screen time is a type of unfavorable environment factor. Thus, we speculate that a longer screen time resulted in shorter play time, shorter companionship time with caregivers and shorter interaction time, resulting in worse social behavior and more obvious autism symptoms. However, this interaction requires further exploration.

### Screen Time Was Related to the DQs of the Children With ASD

The screen time was related to the development of language in the children with ASD in this study. The younger the age and the longer the screen exposure time, the more serious the impact on language development. Hermawati reported that early exposure to electronic media in early life (an age < 2 years) had a negative impact on language (38). Chonchaiya et al. found that children who started watching TV as early as 12 months and watched TV for more than 2 h a day were six times more likely to have language delays. This finding is consistent with our research results. Our Spearman rank correlation results showed that the screen time was negatively correlated with the language DQ in the children with ASD; the longer the screen time, the lower the language DQ. This result was also observed in the ASD subgroup with a longer screen time and the subgroup with a younger age, indicating that the screen time was strongly related to the language DQ.

The screen time was related to not only the language DQ but also the other DQs. A longer screen time restricts the development of physical activities and gross motor ability and limits the development of toy operation ability, which is related to fine motor ability, adaptive behaviors and cognitive levels. Dadson reported that the screen time affected fine motor ability and visual-motor integration. Playing with toys and using object substitution in play can offset these effects to some degree (39). The Canadian Society for Exercise Physiology recommended that for the development of motor skills and cognitive skills, young infants and toddlers should minimize the time spent sitting for a long time (40). One of the most important aspects of sedentary behavior is screen time. It is recommended that children aged under 2 years avoid contact with electronic products to limit this contact as much as possible (40). A longer screen time also limits the development of social skills, further affecting cognitive ability as feedback since the development of children's cognition is derived more from social interaction, family education and parent-child games than from electronic screens and video animation. Previous studies concluded that screen time was unfavorably associated with social skills throughout early childhood (41), which is a finding further emphasized in our study. We found that the screen time was negatively correlated with the other DQs in the longer screen time subgroup and younger age subgroup. Obviously, the younger the age, the more the development level is affected by external factors. We conducted only a simple preliminary investigation of the correlation between the screen time and DQs, and this result needs to be replicated in further studies with larger sample sizes.

## Limitations

Studies have reported that living in a lower-income household, being a minority, living with parents who have lower education levels and being a male are associated with more screen time in the US (24). However, our study did not match the ASD and TD children based on these factors and instead used only randomly surveyed outpatients to observe these phenomena. Further research should consider the impact of these factors.

We did not categorize the type of screen exposure, such as exposure to cartoons, video games or educational nursery rhymes or programs, in detail; thus, the potential benefits of electronic screens may have been ignored, and these benefits may play a role in future ASD interventions. These interventions may help children limit their screen time to a reasonable duration, but the ability to do so is difficult to ensure.

## Conclusion

The screen time of children with ASD is longer than that of neurotypical children. The screen time is related to autistic symptoms and the DQs of children with ASD. The correlation between the screen time and DQs may be more pronounced in children with ASD who have a longer screen time and younger children with ASD. Whether reducing the screen time has positive effects on autism-like symptoms and DQs in children with ASD requires further investigation.

## Data Availability Statement

The raw data supporting the conclusions of this article will be made available by the authors, without undue reservation.

## Ethics Statement

Written informed consent was obtained from the individual(s), and minor(s)' legal guardian/next of kin, for the publication of any potentially identifiable images or data included in this article.

## Author Contributions

H-YD: investigation, writing—original draft, and project administration. BW: revised the manuscript and data analysis. H-HL: conceptualization and methodology. X-JY: investigation. F-YJ: writing—review and editing, supervision, and funding acquisition. All authors contributed to the article and approved the submitted version.

## Conflict of Interest

The authors declare that the research was conducted in the absence of any commercial or financial relationships that could be construed as a potential conflict of interest.
